# Performance of Handwriting and Digital Typing After Carpal Tunnel Release: The TACTUS (Typing Ability in Carpal Tunnel Syndrome) Study

**DOI:** 10.3390/jfmk11030281

**Published:** 2026-07-21

**Authors:** Lorenzo Alirio Diaz Balzani, Guglielmo Torre, Erika Albo, Benedetta Tirone, Giovanna Stelitano, Giulia De Marco, Chiara Capperucci, Rocco Papalia

**Affiliations:** 1 Fondazione Policlinico Universitario Campus Bio-Medico, Via Alvaro del Portillo, 200, 00128 Rome, Italy; l.diaz@policlinicocampus.it (L.A.D.B.); e.albo@policlinicocampus.it (E.A.); giulia.demarco@unicampus.it (G.D.M.); r.papalia@policlinicocampus.it (R.P.); 2 Research Unit of Orthopaedic and Trauma Surgery, Department of Medicine and Surgery, Università Campus Bio-Medico di Roma, Via Alvaro del Portillo, 21, 00128 Rome, Italy; 3 Villa Stuart Sport Clinic, FIFA Medical Centre of Excellence, 00135 Rome, Italy; guglielmo.torre@gmail.com; 4 Department of Movement, Human and Health Sciences, University of Rome “Foro Italico”, 00135 Rome, Italy; 5 University Department of Hand Surgery & Rehabilitation, San Giuseppe Hospital, IRCCS MultiMedica Group, 20123 Milan, Italy; gio.stelitano@gmail.com

**Keywords:** carpal tunnel syndrome, carpal tunnel release, mobile typing, keyboard typing, handwriting

## Abstract

**Background:** Carpal tunnel release (CTR) surgery may have an impact on the speed and accuracy of handwriting and digital typing using a computer or mobile devices. **Methods:** In this prospective cohort study, patients undergoing CTR surgery of the dominant hand, between 18 and 70 years of age, with frequent use of a QWERTY keyboard and smartphone were included. A baseline Visual Analog Scale (VAS) for subjective alteration of writing and Quick Disabilities of the Arm, Shoulder and Hand (QuickDASH) were collected at a maximum 3-month follow-up. Digital typing speed (word per minute, wpm) and accuracy (number of mistakes) were tested. Handwriting was assessed by means of direct supervision of one investigator. **Results:** Of the 30 enrolled patients, 23 (76.7%) completed the 3-month follow-up and were included in the final analysis (mean age 55 ± 12.3 years). Pre- and post-surgery improvements in keyboard typing speed (14.8 ± 6.8 wpm to 17.6 ± 5 wpm) and mobile texting speed (16.7 ± 5.9 wpm to 21.7 ± 6.5 wpm) were significantly improved over time. Accuracy improved significantly only in keyboard typing, where the mean number of errors was reduced (13.1 ± 8.2 to 9.9 ± 5.6). QuickDASH scores decreased significantly (39.1 ± 9.1 to 17 ± 6). **Conclusions:** CTR surgery was associated with improved typing speed and reduced the number of errors (19% and −24%, respectively) as well as texting speed (30%). This improvement may be relevant in daily and occupational activities, as reported in the previous literature.

## 1. Introduction

Carpal tunnel syndrome (CTS) is the most common peripheral nerve entrapment syndrome worldwide, affecting 3.8% of the general population with an annual incidence of 1.5 per 1000 in women and 0.5 per 1000 in men [1,2,3]. Carpal tunnel release surgery is considered the gold standard of treatment for patients who report symptoms that directly impair work and daily activity or compromise nocturnal sleep, for which one or more courses of non-surgical treatment have failed to control symptoms [3]. While the etiology of CTS is usually unknown, there are a number of factors associated with CTS, including work-related biomechanical risk factors, that may contribute to its high socio-economic impact [2,4]. CTS is acknowledged as a work-related condition in some countries, including Italy. People with diagnosed CTS have more absences from work than people without this diagnosis [5,6,7,8].

With progression, nerve compression may affect the ability to perform fine motor tasks that require the thumb and index finger, especially precision pinch movements, thus reducing dexterity [9,10,11,12]. This has a negative effect on quality of life by compromising the proper performance of many daily life and work-related activities, including writing [11,13]. Handwriting requires extreme precision, proper cutaneous sensation, stability, and coordination of the fingers [14]. Keyboard typing is also a complex activity where wrist-hand movements and hand kinematics play a central role [15,16,17]. A recent study suggested that, provided that both hands are used for typing, manual coordination processes typical of touch-typing are the same in phone texting [18]. Furthermore, the growing use of phone texting from smartphones requires repetitive thumb motion and a grip force exerted by the other fingers that may increase the impact of CTS symptoms on daily and job activities, especially flexor tendon thickening, inflammation, and swelling [19]. It has been reported that phone texting activity is compromised in patients with CTS when compared to healthy volunteers [19].

Rabin and Gordon [20] showed that tactile feedback contributes to consistency of finger movements during touch-typing. A more recent investigation indicated that keyboard typing speed returned to preoperative levels between two and three weeks after carpal tunnel release, improving beyond preoperative levels [21]. Regarding handwriting, CTS is known to have a negative impact on performance that then improves after surgical release [22].

Given the growing importance of digital texting and the still relevant role of handwriting in everyday life and work activities, we performed a prospective investigation to analyze improvement in combined writing performance (handwriting, keyboard typing, and phone texting) after open surgical release.

## 2. Materials and Methods

### 2.1. Study Design and Setting

The study was designed as an observational prospective cohort investigation. Between January and June 2021, a total of 150 patients underwent carpal tunnel release surgery at our institution, a tertiary orthopedic center in Italy. Of these, 30 patients met the predefined eligibility criteria and were recruited consecutively for inclusion in the study (see Table 1). The study was approved by the local ethics committee. All patients signed an informed consent form before being recruited for the investigation. The STROBE checklist was used to report the results of the study.

### 2.2. Inclusion and Exclusion Criteria

Inclusion criteria for recruitment were age between 18 and 70 years; diagnosis of CTS in the dominant hand (or the hand they use to write); being a candidate for open carpal tunnel release surgery, based on persistent or worsening symptoms despite non-operative treatment, including splinting or injections, and confirmed by clinical findings; positive electromyography for impaired median nerve conduction velocity (moderate or severe) in the affected limb [23]; frequent use of a QWERTY keyboard (defined as more than three times per week); and use of a smartphone on a daily basis for communication and recreational purposes. Exclusion criteria were not speaking or comprehension of the Italian language; use of electronic typing (PC or mobile) fewer than two times per week; recurrent CTS previously treated surgically; presence of other concurrent compressive neuropathies in the same limb; concomitant psychiatric disorders; and illiteracy (defined as the inability to read and write).

### 2.3. CTS Diagnosis and Baseline Assessment

Clinical diagnosis of CTS was made using the Tinel test, Phalen test and pinch grip test, where at least two tests were positive. In addition, all patients underwent clinical assessment of sensory and motor function. Preoperatively, all reported reduced sensation or paresthesia in the median nerve distribution, often associated with impaired fine motor control of the thumb, index, and middle fingers, consistent with the sensory-motor deficits typically observed in carpal tunnel syndrome. The diagnosis was confirmed through electromyography, carried out by a trained neurologist at our institution, reporting moderate to severely impaired conduction velocity of the median nerve [3]. The clinical examination was carried out by a fellowship-trained hand surgeon (LDB) at the time of presentation to the clinic. The examination was repeated by the same surgeon and a resident at the time of surgery (immediately pre-operatively). Although recent evidence supports the complementary role of ultrasonography in CTS diagnosis [4] at the time of our patient recruitment, ultrasonography had not yet become routine clinical practice in our institution.

After completing recruitment procedures, patients were evaluated for demographic parameters, specifically age, sex, affected limb (right or left hand), education level, comorbidities, and weekly frequency of use of handwriting or digital typing (low ≤ 3 days per week; moderate = 3–5 days per week; high ≥ 5 days per week). The study population was not specifically selected based on typing-intensive occupations but on regular use of a QWERTY keyboard and smartphone in daily life. An evaluation of subjective alterations of writing performance was asked of each patient at the baseline compared to their usual pre-CTS ability using a 0–10 Visual Analog Scale (VAS) (0 = patient’s writing was completely unaffected by the CTS condition; 10 = unable to write). Although difficulty in mobile typing was not always the primary complaint, it was assessed systematically due to its functional importance. Patients also compiled the Italian version of the Quick Disabilities of the Arm, Shoulder and Hand (QuickDASH) questionnaire [24]. The questionnaire was administered according to its standard instructions, reflecting the patient’s current functional status at each assessment.

### 2.4. Handwriting and Digital Typing Tasks

Writing performance tests were then performed under the supervision of one investigator (B.T.). Blinding of the investigator was not feasible because of the prospective design and repeated follow-up assessments. For testing handwriting speed and accuracy, the patient was asked to use a standard ballpoint pen and copy on a piece of white paper a paragraph of 81 words (498 characters) that was presented on a PC screen in Times New Roman, 11-point font. Handwriting errors were defined as corrections requiring cancelation and rewriting of the text. Handwriting speed was expressed as words per minute (WPM). During the task, the investigator used a stopwatch to measure the time needed to complete the task and checked the occurrence of writing mistakes that led the patients to cancel or modify (cancel and re-write) the written text. To test digital typing, a dedicated webpage in Google Colab was prepared (Python 3.5 coding language) by one of the investigators (G.T.). The webpage displayed two paragraphs, each with a free text space; every patient used the appropriate keyboard to retype the displayed text into the free text space. The webpage was accessed by a personal computer, and the first paragraph (65 words, 450 characters) was copied using a QWERTY PC keyboard. Next, the page was loaded on the patient’s own touch-screen smartphone, and the second paragraph (71 words, 461 characters) was copied using the QWERTY keyboard of the smartphone. Participants used their own smartphones to reproduce their usual texting environment and maximize ecological validity. Device characteristics and keyboard settings were not standardized. At the end of each task, the Colab script automatically calculated typing speed (words per minute, WPM) and accuracy by comparing the final transcribed text with the reference text. Any discrepancy between the reference and submitted text, including omitted, added, or incorrectly typed characters or words, was counted as an error. Corrections made before final submission were not recorded as errors. Once the webpage was loaded in the browser, the use of the page was not affected by internet connection speed, except for the calculation of times and errors (that had no influence on typing speed and accuracy).

An ergometric position was established for PC typing. Each patient sat in front of the screen on an office chair with adjustable arms. The forearms and elbows were lying on the desk, towards the keyboard. The same position was used to perform the smartphone task: open the dedicated webpage in the browser of their mobile phone and perform the second copy task by gripping the device with the long fingers of both hands and texting with the two thumbs. For the handwriting task, participants were asked to copy the text using their usual speed and accuracy.

### 2.5. Surgical Procedure and Postoperative Management

All surgical procedures were performed under local anesthesia using an open technique through a longitudinal palmar incision. Dissection was carried out under direct visualization, and complete release of the transverse carpal ligament was achieved. Patients were instructed to elevate the hand to chest level and to perform small movements of the fingers in the first week. After removal of stitches at 10 to 14 days postoperatively, depending on wound healing, careful and progressive recovery of full finger and wrist range of motion was allowed.

### 2.6. Follow-Up Assessment

After surgery, patients were followed for three months, with six post-surgery evaluations: weeks one, two, and three, followed by monthly evaluations at months one, two, and three after surgery. During these six visits, patients performed the writing tests and the QuickDASH and were interviewed for subjective evaluations.

### 2.7. Data Management and Processing

After the dataset was checked for quality and validation, missing data were less than 10% of the series and were not imputed, given the small size of the sample. To use categorical variables in multivariate analysis of variance, a numeric discrete non-ordinal transformation was carried out. Patients lost to follow-up were excluded from the longitudinal analyses (complete-case analysis). Baseline characteristics of completers and non-completers were compared to evaluate the potential risk of attrition bias. No data imputation was performed.

### 2.8. Statistical Analysis

Continuous variables were reported as mean and standard deviation; binary or categorical variables were reported as percentages; discrete variables were reported as median and interquartile range (IQR). Normality of speed and accuracy variables was assessed before analysis, and sphericity was evaluated using Mauchly’s test. When the assumption of sphericity was violated, appropriate corrections were applied. One-way ANOVA for repeated measures was used to analyze changes in speed (wpm) and accuracy (number of errors). Sample size was calculated using a previous study as reference [21], where, after 12 postoperative weeks, touch-typing increased 4.3 wpm (from 49.2 ± 2.7 to 53.5 ± 3.5 wpm; +8.7%). Power was calculated with the following parameters: smallest effect of interest 4.3; effect size 1.35; alpha 0.05; 1-beta 0.9 (90% power); a minimum sample size of 10 patients was determined. To avoid bias due to patients lost at follow-up, a threshold of a minimum of 15 patients was set. Given the discrepancy in baseline typing speed between the reference study and our cohort, a post hoc power analysis was performed for the primary outcome. Using a repeated-measures ANOVA (effect size f = 0.30, α = 0.05, n = 23, seven measurements), the achieved statistical power was 97.9%.

## 3. Results

### 3.1. Patient Demographics

A total of 30 patients met our inclusion criteria. Seven patients (23%) were lost to follow-up (Supplementary Figure S1), leaving twenty-three patients (mean age 55 ± 12.3 years) for the final analysis. Comparison of baseline demographic and clinical characteristics between patients who completed follow-up and those lost to follow-up did not reveal clinically relevant differences. Demographic information is summarized in Table 1. Of the 23 patients, 21 were righthanded and 2 were ambidextrous. Participants included both clerical and manual workers. Education level, comorbidities, and writing frequency are presented in Table 1 and Table 2.

### 3.2. Writing and Typing Performance

**Figure 1:**
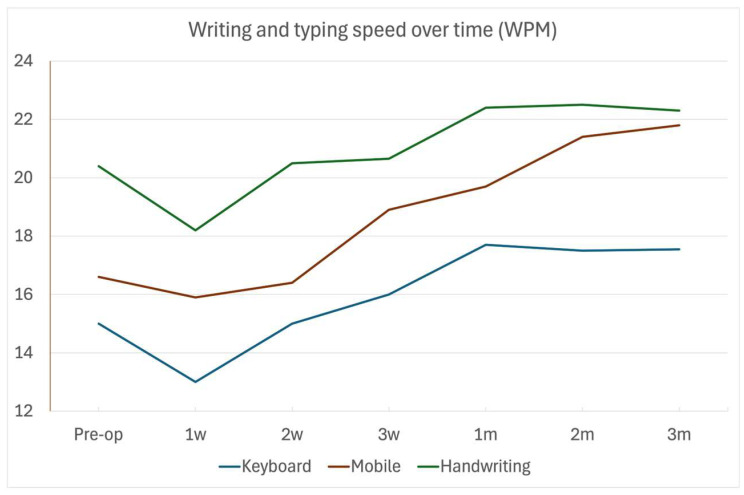
Variation in writing speed over time.

**Figure 2:**
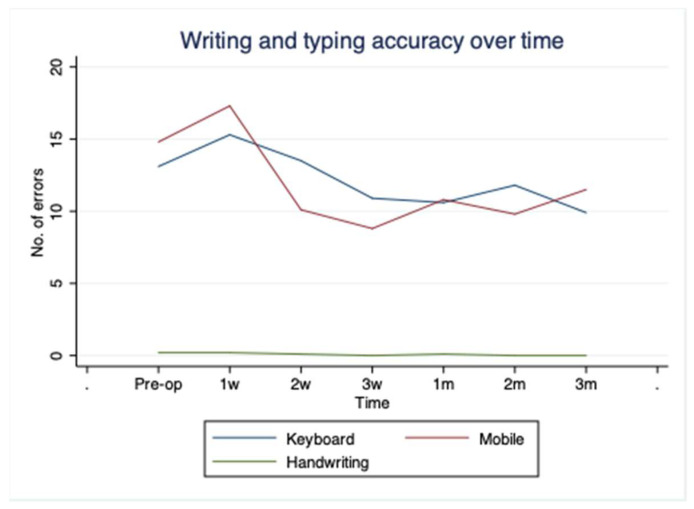
Variation in writing accuracy over time.

The median preoperative VAS for subjective impairment during writing and typing tasks was 6 (IQR 4–8). VAS was collected only at baseline to assess patients’ perceived functional impairment and was therefore not included in the longitudinal analyses. Variation in speed (wpm) and accuracy (number of errors) over time for each type of writing—handwriting, PC keyboard typing, and mobile texting—showed a trend of improvement after surgery. Pre-operative values were exceeded between two and three weeks after surgery (Table 3). A statistically significant improvement was seen for keyboard typing speed (14.8 ± 6.8 wpm preoperatively to 17.6 ± 5 wpm at last follow-up; *p* < 0.001) and the mobile texting speed (16.7 ± 5.9 wpm to 21.7 ± 6.5 wpm; *p* < 0.001) (Figure 1). The observed effect size for the primary outcome was small (Cohen’s d = 0.29). Based on the predefined minimal clinically important difference (MCID) of 4 WPM used for sample size calculation, 5/12 patients (41.7%) achieved a clinically meaningful improvement in keyboard typing speed at the final follow-up. Similarly, the QuickDASH scoring decreased significantly, from 39.1 ± 9.1% to 17 ± 6% (*p* < 0.001) (see Table 3 for details). Typing accuracy improved significantly (*p* = 0.036) only in keyboard typing; the mean number of errors was reduced from 13.1 ± 8.2 (pre-operative) to 9.9 ± 5.6 (final follow-up) (Figure 2).

All were able to complete the tests, although some showed limitations in early performance due to postoperative discomfort.

No major complications were observed that would have required revision surgery, such as infection, compressive hematoma, or delayed wound healing. Minor complaints such as scar sensitivity or transient discomfort were not systematically recorded, as they were considered beyond the scope of this functional performance study.

## 4. Discussion

Our results showed that digital typing ability in patients affected by CTS was significantly improved after carpal tunnel release surgery. The speed and accuracy of typing on a PC keyboard and the speed of mobile texting improved over the 3-month post-operative study period (Table 4). Trends of speed improvement showed a steep increase during the first post-operative month that plateaued between one and three postoperative months (Figure 1). While typing performance reached a plateau after the first postoperative month, QuickDASH scores continued to improve, likely reflecting broader recovery of upper limb function beyond typing ability.

There are several studies that have investigated dexterity deterioration caused by CTS. Most studies focused on hand-wrist coordination and on grip and pinch movements and showed that CTS leads to a reduced precision and increased variability of these motor tasks [10,12,25]. Similar parameters have been reported after corrective surgery to check the functional recovery of the patients [26].

Recent work has evaluated a patient’s functionality before and after release surgery using more specific motor tasks, including tasks related to writing. Kuo et al. [22] evaluated a number of parameters connected to handwriting (hand dexterity, pen tip force) and reported significant performance improvements after surgery for CTS. In another study, Zumsteg et al. [21] assessed keyboard typing speed and found results that parallel our own. Their data showed a significant reduction in writing speed during the first week after surgery that returned to preoperative levels by the second and third postoperative weeks. A progressive trend towards improvement in performance continued until the last follow-up visit (postoperative week 12). Compared with the study by Zumsteg et al. [22]., our cohort showed a smaller absolute improvement in typing speed but a greater relative improvement. This difference is likely explained by the substantially lower baseline typing speed of our patients, reflecting differences in baseline typing proficiency and study population characteristics rather than postoperative recovery alone. Although the mean improvement did not reach the predefined MCID of 4 WPM, a substantial proportion of patients achieved this threshold. Therefore, the observed improvements should be interpreted relative to each patient’s baseline functional performance rather than occupational typing standards.

Our study also evaluated the use of both keyboards and mobile-writing (“texting”) evaluations. In addition, we assessed typing speed and accuracy during a bilateral task even though only one hand underwent surgery. Our results, and the previous findings from Zumsteg et al. [21], suggest that CTS compromises typing skills and support the importance of including a typing assessment in the clinical evaluation of the patient affected by CTS.

The heterogeneous occupational background of the cohort may limit the generalizability of our findings to younger populations or individuals whose occupations require intensive keyboard use. Given the limited sample size, subgroup analyses should be considered exploratory and interpreted with caution. Furthermore, subgroup analyses according to electrophysiological severity were not performed because stratification into moderate and severe CTS would have resulted in very small subgroups, precluding meaningful statistical comparisons. Finally, the 3-month follow-up was designed to evaluate early postoperative recovery and may not fully capture long-term functional outcomes. Longer follow-up is warranted to determine the durability of the observed improvements.

While we did not observe any significant change in accuracy for handwriting, all other parameters showed an initial deterioration one week after surgery, followed by a gradual improvement over time, leading to a return to pre-operative levels within 2–3 weeks after surgery. A trend towards an increase in performance from baseline was observed, although only the QuickDASH scores and mobile typing speed showed significantly different values at the last follow-up visit. Early post-operative performance (week 1) often remained below baseline levels, with subsequent recovery typically occurring between two and three weeks post-surgery.

CTS particularly affects mobility and sensibility of the thumb, index, and middle finger, leading to a deterioration of dexterity and writing activities. While keyboard and mobile typing require the same type of coordination [18], keyboard typing involves all fingers, while mobile writing involves mainly the index finger and the thumb. These considerations are in line with our objective speed measurements, suggesting that mobile writing might be the most affected typing activity in CTS patients. The handwriting assessment likely showed a floor effect, as very few errors were observed at baseline. Although the task was designed to reproduce a standardized everyday writing activity, more demanding assessments evaluating legibility, letter formation, or timed writing may be more sensitive to detect subtle CTS-related impairment and postoperative recovery. Moreover, digital typing may be more affected than handwriting because it requires rapid, repetitive, and highly coordinated finger movements.

Unlike general functional scores such as QuickDASH, assessing digital typing provides task-specific insight into real-world activities impacted by CTS, such as occupational keyboard use or texting, which may be highly relevant in today’s digital work environment.

While scores such as the QuickDASH provide a validated overview of upper limb function, typing tasks can reflect task-specific limitations in daily activities such as texting or computer use, which may be particularly relevant for digitally active patients.

Our study presents some relevant limitations. We did not enroll a control group of patients without CTS to compare their performance to that of the CTS patients. The inclusion of a control group could have helped assess normative values to which to compare post-operative results to understand whether after surgery writing and typing performance would not only improve but also correspond to those of healthy patients. Furthermore, practicing the same script over follow-up could be a relevant confounding factor to assess actual improvement of typing speed. Repeated testing may have introduced a practice effect, potentially contributing to improved performance through task familiarization. However, baseline performance was likely impaired by pain, paresthesia, and reduced dexterity related to carpal tunnel syndrome. Moreover, the parallel improvement in QuickDASH scores and the recovery pattern observed suggest that postoperative functional recovery, rather than practice alone, was the main contributor to the observed gains. Future studies should include a habituation session or a non-operative control group to better distinguish recovery from learning effects. Finally, correlations between subjective perception of writing difficulty and objective typing performance were not investigated and should be evaluated in larger studies.

In addition, our tests revolved around speed and accuracy of writing over a short period of time (a few minutes) and did not assess how CTS and surgery might affect endurance in tasks that might be more relevant, especially in certain occupational settings.

Using participants’ own smartphones increased ecological validity but may have introduced device-related variability. Finally, blinding of the handwriting assessor was not feasible, which may have introduced a small observer bias in the manually timed handwriting task, although the primary typing outcomes were automatically recorded by the software. Although the original sample size calculation was based on a previous study including participants with substantially higher baseline typing speed, the post hoc power analysis demonstrated adequate statistical power for the primary outcome. Nevertheless, the relatively small sample size and the observational design warrant cautious interpretation of the findings.

Finally, the relatively low baseline typing proficiency of our cohort may limit the generalizability of these findings to younger populations or professional touch typists. Although approximately one-quarter of patients were lost to follow-up, no clinically relevant baseline differences were observed between completers and non-completers, suggesting a limited risk of attrition bias. Nevertheless, attrition may have reduced the generalizability of our findings.

From our results, we can conclude that CTS impairs writing abilities that improve rapidly after surgery, especially for mobile typing and general dexterity. We suggest this outcome might be related to the improvement in thumb mobility as well as improvements in sensibility. From a clinical practice perspective, these results further highlight the beneficial effects of CTS release on typing ability, information that the surgeon may discuss with patients during the acquisition of the informed consent, and underline the impact of the surgical procedure on a patient’s occupation.

## Figures and Tables

**Table 1:** Patient demographics.

Number of patients, N	23
Sex N (%)	F = 18 (78%); M = 5 (22%)
Age (Mean ± SD)	56 ± 12
Writing difficulty (1 to 10; Mean ± SD)	6.21 ± 2.52
Highest level of education	
Primary school	2 (9%)
Middle school	6 (26%)
High school	11 (48%)
University	4 (17%)
Co-morbidities	
Diabetes	2 (8.7%)
Metabolic syndrome	3 (13%)
Dysthyroidism	6 (26.1%)

F = female; M = male.

**Table 2:** Writing frequency.

Frequency	Keyboard Typing	Mobile Texting	Handwriting
High, N (%)	6 (26%)	17 (74%)	5 (22%)
Moderate, N (%)	4 (17%)	1 (4%)	8 (35%)
Low, N (%)	13 (57%)	5 (22%)	10 (43%)

N = number.

**Table 3:** Means (SD) of speed, accuracy and QuickDASH score over time.

	Baseline	1w	2w	3w	1m	2m	3m	*p*=
**Keyboard typing**								
Speed (wpm)	14.8 (6.8)	13.4 (4.5)	14.9 (5.2)	16.1 (5.6)	17.8 (7.2)	17.5 (6.4)	17.6 (5.0)	0.0004 *
Errors (#)	13.1 (8.2)	15.3 (7.8)	13.5 (8.3)	10.9 (5.9)	10.6 (7.2)	11.8 (6.4)	9.9 (5.6)	0.036 *
**Mobile texting**								
Speed (wpm)	16.7 (5.9)	15.8 (4.9)	16.5 (5.5)	18.9 (5.9)	19.7 (6.7)	21.2 (6.5)	21.7 (6.5)	0.0001 *
Errors (#)	14.8 (14.7)	17.3 (16.1)	10.1 (5.8)	8.8 (5.6)	10.8 (12.8)	9.8 (7.7)	11.5 (14.7)	0.127
**Handwriting**								
Speed (wpm)	20.5 (7.1)	18.3 (7.3)	20.7 (5.9)	20.9 (5.1)	22.7 (6)	22.8 (7)	22.4 (4.0)	0.063
Errors (#)	0.2 (0.6)	0.2 (0.5)	0.1 (0.7)	0 (0)	0.1 (0.2)	0 (0)	0 (0)	0.375
**QuickDASH**								
QuickDASH Score	38.9 (9.1)	38.3 (7.5)	33.8 (9.1)	30.8 (7.5)	29.2 (6.1)	24.1 (9.1)	17 (5.7)	<0.001 *

SD = standard deviation; w = week, m = month. * = statistically significant.

**Table 4:** Table of significance levels for two-way ANOVA interactions.

	Keyboard Typing Speed (wpm)	Mobile Texting Speed (wpm)	Handwriting Speed (wpm)
Male sex	*p* = 0.997	*p* = 0.851	*p* = 0.974
Diabetes	-	-	-
Metabolic syndrome	-	-	-
Dysthyroidism	-	-	-
Writing frequency	*p* = 0.991	*p* = 0.905	*p* = 0.990
Education level	*p* = 0.999	*p* = 0.929	*p* = 0.999

Empty = too few observations in that subgroup for meaningful analysis.

## Data Availability

The original contributions presented in this study are included in the article/Supplementary Material. Further inquiries can be directed to the corresponding author.

## Supplementary Materials

The following supporting information can be downloaded at: https://www.mdpi.com/article/10.3390/jfmk11030281/s1. Figure S1: Flow diagram of participant recruitment, follow-up, and inclusion in the final complete-case analysis.

## References

[1] Aboonq M.S. (2015). Pathophysiology of carpal tunnel syndrome. Neurosciences.

[2] Newington L., Harris E.C., Walker-Bone K. (2015). Carpal tunnel syndrome and work. Best Pract. Res. Clin. Rheumatol..

[3] Padua L., Coraci D., Erra C., Pazzaglia C., Paolasso I., Loreti C., Caliandro P., Hobson-Webb L.D. (2016). Carpal tunnel syndrome: Clinical features, diagnosis, and management. Lancet Neurol..

[4] Chen J., Fowler J.R. (2023). Comparison of diagnostic accuracy of electrodiagnostic testing and ultrasonography for carpal tunnel syndrome. Hand.

[5] Kozak A., Schedlbauer G., Wirth T., Euler U., Westermann C., Nienhaus A. (2015). Association between work-related biomechanical risk factors and the occurrence of carpal tunnel syndrome: An overview of systematic reviews and a meta-analysis of current research. BMC Musculoskelet. Disord..

[6] Atroshi I., Zhou C., Jöud A., Petersson I.F., Englund M. (2015). Sickness absence from work among persons with new physician-diagnosed carpal tunnel syndrome: A population-based matched-cohort study. PLoS ONE.

[7] Cowan J., Makanji H., Mudgal C., Jupiter J., Ring D. (2012). Determinants of return to work after carpal tunnel release. J. Hand Surg..

[8] Katz J.N., Lew R.A., Bessette L., Punnett L., Fossel A.H., Mooney N., Keller R.B. (1998). Prevalence and predictors of long-term work disability due to carpal tunnel syndrome. Am. J. Ind. Med..

[9] Wang A.A., Hutchinson D.T., Vanderhooft J.E. (2003). Bilateral simultaneous open carpal tunnel release: A prospective study of postoperative activities of daily living and patient satisfaction. J. Hand Surg..

[10] Padua L., Cuccagna C., Giovannini S., Coraci D., Pelosi L., Loreti C., Bernabei R., Hobson-Webb L.D. (2023). Carpal tunnel syndrome: Updated evidence and new questions. Lancet Neurol..

[11] Gehrmann S., Tang J., Kaufmann R.A., Goitz R.J., Windolf J., Li Z.M. (2008). Variability of precision pinch movements caused by carpal tunnel syndrome. J. Hand Surg..

[12] Levine D.W., Simmons B.P., Koris M.J., Daltroy L.H., Hohl G.G., Fossel A.H., Katz J.N. (1993). A self-administered questionnaire for the assessment of severity of symptoms and functional status in carpal tunnel syndrome. J. Bone Jt. Surg..

[13] Nataraj R., Evans P.J., Seitz W.H., Li Z.M. (2014). Pathokinematics of precision pinch movement associated with carpal tunnel syndrome. J. Orthop. Res..

[14] Jerosch-Herold C., Mason R., Chojnowski A.J. (2008). A qualitative study of the experiences and expectations of surgery in patients with carpal tunnel syndrome. J. Hand Ther..

[15] Ebied A.M., Kemp G.J., Frostick S.P. (2004). The role of cutaneous sensation in the motor function of the hand. J. Orthop. Res..

[16] Baker N.A., Cham R., Cidboy E.H., Cook J., Redfern M.S. (2007). Kinematics of the fingers and hands during computer keyboard use. Clin. Biomech..

[17] Dennerlein J.T., Mote C.D., Rempel D.M. (1998). Control strategies for finger movement during touch-typing. The role of the extrinsic muscles during a keystroke. Exp. Brain Res..

[18] Rempel D.M., Keir P.J., Bach J.M. (2008). Effect of wrist posture on carpal tunnel pressure while typing. J. Orthop. Res..

[19] Cerni T., Longcamp M., Job R. (2016). Two thumbs and one index: A comparison of manual coordination in touch-typing and mobile-typing. Acta Psychol..

[20] Szekeres M., Cheung D., Macdermid J. (2021). The impact of cell phone texting on superficial blood flow, touch threshold, and symptoms for individuals with carpal tunnel syndrome. Work.

[21] Rabin E., Gordon A.M. (2004). Tactile feedback contributes to consistency of finger movements during typing. Exp. Brain Res..

[22] Zumsteg J.W., Crump M.J., Logan G.D., Weikert D.R., Lee D.H. (2017). The Effect of Carpal Tunnel Release on Typing Performance. J. Hand Surg..

[23] Kuo L.C., Hsu H.M., Wu P.T., Lin S.C., Hsu H.Y., Jou I.M. (2014). Impact of distal median neuropathy on handwriting performance for patients with carpal tunnel syndrome in office and administrative support occupations. J. Occup. Rehabil..

[24] Bland J.D. (2000). A neurophysiological grading scale for carpal tunnel syndrome. Muscle Nerve.

[25] Padua R., Padua L., Ceccarelli E., Romanini E., Zanoli G., Amadio P., Campi A. (2003). Italian version of the Disability of the Arm, Shoulder and Hand (DASH) questionnaire. Cross-cultural adaptation and validation. J. Hand Surg..

[26] Nataraj R., Evans P.J., Seitz W.H., Li Z.M. (2014). Effects of carpal tunnel syndrome on reach-to-pinch performance. PLoS ONE.

