# Supplementary material for: Performance of Handwriting and Digital Typing After Carpal Tunnel Release: The TACTUS (Typing Ability in Carpal Tunnel Syndrome) Study
Source: J Funct Morphol Kinesiol. 2026 Jul 21;11(3):281. doi: 10.3390/jfmk11030281 (PMC13398077; doi:10.3390/jfmk11030281)
Supplement: Supplementary file 1 [file jfmk-11-00281-s001.zip › jfmk-4375315-supplementary.pdf]

Supplementary Materials

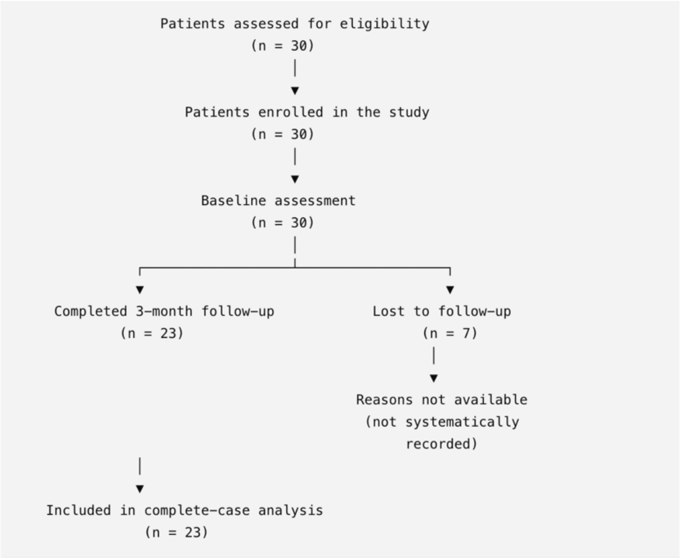

Supplementary Figure S1. Flow diagram of participant recruitment, follow-up, and inclusion in the final complete-case analysis.
